# Pharmacological treatment options for binge-eating disorder: a review

**DOI:** 10.1192/j.eurpsy.2026.11371

**Published:** 2026-07-31

**Authors:** R. M. S. Lousada, R. Nogueira, D. Cotovio

**Affiliations:** Hospital Beatriz Angelo, Lisbon, Portugal

## Abstract

**Introduction:**

Binge-eating disorder (BED) is the most common eating disorder and is associated with significant psychiatric comorbidity and obesity. While psychotherapy (especially cognitive behavioural therapy) remains first-line, pharmacotherapy is often used when psychotherapy is unavailable or as adjunctive treatment.

**Objectives:**

The aim of this review is to explore the published literature addressing the pharmacological treatments for BED.

**Methods:**

A non-systematic literature review was conducted using PubMed and Google Scholar databases. The following search terms were used: “binge eating disorder” and “pharmacological treatments”.

**Results:**

Lisdexamfetamine (LDX) is the only FDA-approved pharmacotherapy for the treatment of moderate to severe binge eating disorder (BED) in adult patients. It demonstrates reductions in binge frequency and higher remission rates versus placebo. SSRIs (notably fluoxetine and sertraline) show moderate benefit for reducing binge frequency and associated depressive symptoms, though weight change is usually negligible. Topiramate has demonstrated efficacy in clinical trials (often with weight loss) but is limited by cognitive and paresthesia adverse effects. Combination naltrexone-bupropion and other anti-obesity agents have mixed evidence. GLP-1 receptor agonists (e.g., semaglutide) are emerging as potential options for reducing food cravings and compulsive eating, though data are limited.

**Conclusions:**

LDX and several off-label options (SSRIs, topiramate) have the strongest evidence for symptomatic reduction in BED; newer metabolic agentes, such as GLP-1 receptor agonists and naltrexone–bupropion, are promising but need BED-targeted studies. Future research is needed to compare pharmacotherapy head-to-head and examine long-term outcomes.

**Disclosure of Interest:**

None Declared

